# Effects of C-type natriuretic peptide on meiotic arrest and developmental competence of bovine oocyte derived from small and medium follicles

**DOI:** 10.1038/s41598-020-75354-5

**Published:** 2020-10-26

**Authors:** Zhenwei Jia, Xueli Wang

**Affiliations:** College of Animal Science and Technology, Inner Mongolia University for the Nationalities, 536 West Huolinhe Street, Tongliao, 028000 Inner Mongolia People’s Republic of China

**Keywords:** Cell biology, Developmental biology

## Abstract

The present study aimed to evaluate the effects of C-type natriuretic peptide (CNP) on meiotic arrest and developmental competence of bovine oocyte derived from follicles of different sizes. Collected immature cumulus-oocyte complexes from small follicles (< 3 mm) and medium follicles (3–8 mm) were cultured for 6 h in basal medium supplementated without or with 200 nM CNP. We observed that CNP effectively sustained meiotic arrest at germinal vesicle stage in in vitro cultured bovine oocytes from follicles of different sizes. Moreover, CNP treatment significantly improved the levels of cGMP in both cumulus cells and oocytes, as well as the levels of cAMP in oocytes regardless of follicle size. Based on the above results, we tested the effect of a novel in vitro maturation (IVM) system based on CNP-pretreatment, including a pre-IVM phase for 6 h using 200 nM CNP, followed by a extended IVM phase for 28 h, on developmental competence of bovine oocyte derived from small follicles (< 3 mm) and medium follicles (3–8 mm) compared to standard IVM system. The results showed that athough the novel IVM system based on CNP-pretreatment enhanced the developmental potencial of oocytes obtained from large follicles, but had no effect on the developmental comptence of oocytes obtained from small follicles.

## Introduction

It is well established that the developmental capacity of bovine oocytes undergoing in vitro maturation (IVM) is poor^1,2^. The decrease in the developmental competence of in vitro-matured oocytes may be partly due to asynchronous nuclear and cytoplasmic maturation, leading to insufficient cytoplasmic maturity^3^. Thus, synchronization of nuclear and cytoplasmic maturation in antral oocytes arrested at the immature GV-stage is of fundamental importance for successful fertilization and embryo development^4^.

The somatic cell compartment of the antral follicles plays a key role in the maintenance of oocyte meiotic arrest^5,6^. In addition, evidence indicates that oocytes meiotic arrest at the germinal vesicle (GV) stage is associated with the intra-oocyte levels of cAMP and high levels of cAMP within the oocyte maintain meiotic arrest^7–9^. More importantly, C-type natriuretic peptide (CNP), which is expressed at high levels in mural granulosa cells, acts on the receptor natriuretic peptide receptor 2 (NPR2) in cumulus cells to generate cGMP, cyclic GMP then enters the oocyte via gap-junctional communication (GJC) and regulates the levels of cAMP by inhibiting the hydrolyzing activity of specific phosphodiesterase 3A (PDE3A), thus maintaining oocytes meiotic arrest^10,11^. Given the inhibitory role of CNP in the modulation of oocyte meiotic maturation, intensive efforts have focused on the development of two-step culture systems using CNP to delay or temporarily prevent spontaneous nuclear maturation, with the aim of promoting cytoplasmic maturation and subsequently improving bovine oocyte developmental competence in vitro ^11–14^. Notably, a novel IVM system based on CNP-pretreatment, including a pre-IVM phase for 6 h using 200 nM CNP, followed by a extended IVM phase for 26–28 h, has demonstrated a remarkable improvement in bovine oocyte developmental comptence^11^.

Oocyte developmental competence is highly correlated with follicle size^15^. Bovine oocytes from small antral follicles (< 3 mm) have low competence to be matured in vitro and to assure early embryo development^16^. Therefore, bovine oocyte from medium antral follicles (MFs, 3–8 mm) are commonly used for IVM and in vitro embryo production. Consequently, oocytes from small follicles (SFs, < 3 mm) are discarded because of their low developmental competence, resulting in valuable genetic materials being wasted. Thus, the present study aimed to investigate the effects of CNP on meiotic arrest and developmental competence of bovine oocyte derived from follicles of different sizes, with an emphasis on the efficiency of the novel IVM system based on CNP-pretreatment at improving developmental comptence of bovine oocytes from SFs compared with conventional IVM system.

## Results

### Effect of CNP on meiotic arrest in bovine oocytes in vitro

**Figure 1:**
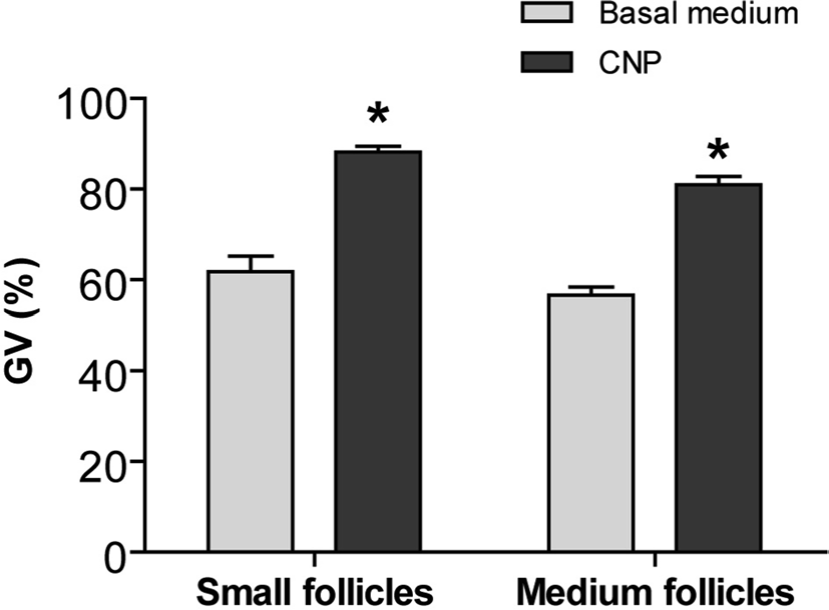
Effect of CNP on meiotic arrest of bovine oocytes cultured in vitro from different sizes follicles. COCs derived from small follicles (SFs) and medium follicles (MFs) were cultured for 6 h in basal medium without or with 200 nM CNP. The rate of oocyte arrested at the GV stage was examined at the end of culture. Graphs show the mean ± SEM values of four independent experiments; The asterisks represent significant differences between the two groups with the same size follicle (*P* < *0.05*). CNP: C-type natriureticpeptide; COCs: cumulus oocyte complexes; GV: germinal vesicle.

To examine wheather CNP can maintain meiotic arrest in bovine oocytes derived from different size follicles, cumulus-oocyte complexes (COCs) from different size follicles were cultured for 6 h in basal medium supplementated without or with 200 nM CNP. We found that COCs treated with CNP displayed a significantly higher rate of GV regardless of follicle size, compared to basal group (no CNP treatment) (*P* < *0.05*) (Fig. 1).

### Effect of CNP on cGMP levels in cumulus cells and oocytes of COCs

**Figure 2:**
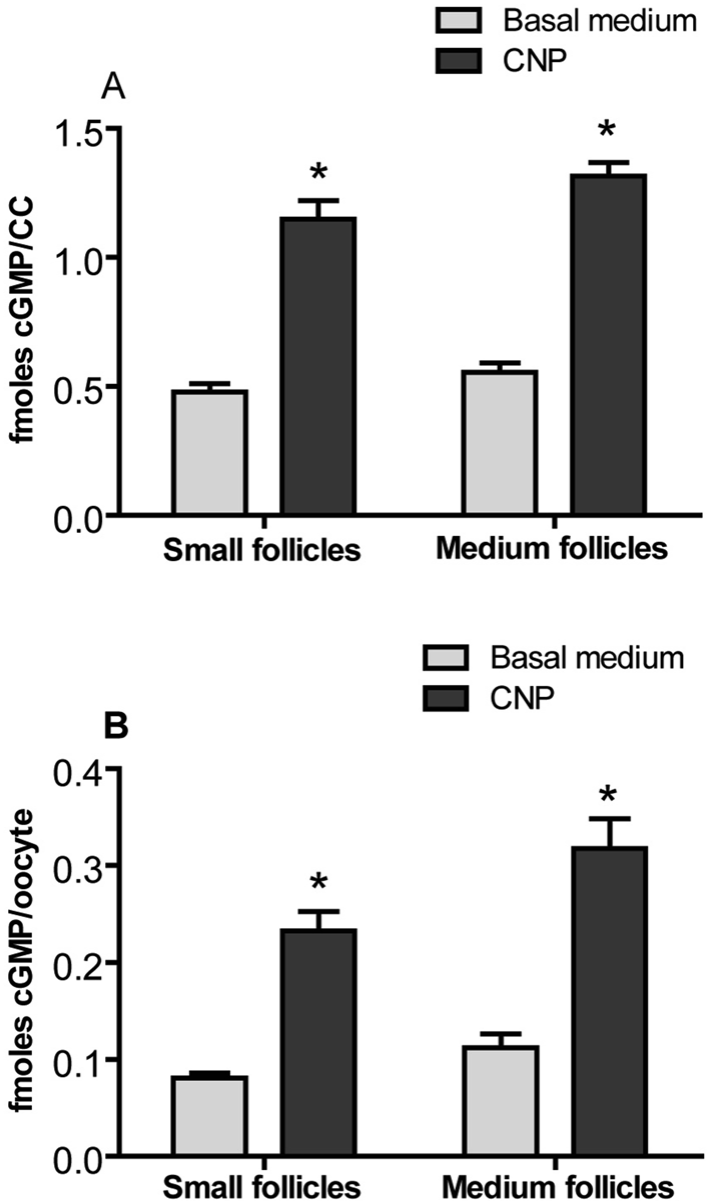
Effect of CNP on the levels of cGMP in bovine cumulus cells and oocytes. COCs derived from small follicles (SFs) and medium follicles (MFs) were cultured for 6 h in basal medium without or with 200 nM CNP. The levels of cGMP in bovine cumulus cells and oocytes were detected at the end of culture. (**A**) The levels of cGMP in bovine cumulus cells. (**B**) The levels of cGMP in bovine oocytes. Graphs show the mean ± SEM values of four independent experiments; The asterisks represent significant differences between the two groups with the same size follicle (*P* < *0.05*).

To determine whether CNP can stimulate COCs from follicles of different sizes to generate cGMP, COCs from SFs and MFs were cultured for 6 h in basal medium supplementated without or with 200 nM CNP. The results showed that the levels of cGMP in both cumulus cells and oocytes regardless of follicle size were significantly higher in CNP treatment group than that in basal medium group (no CNP treatment) (*P* < *0.05*) (Fig. 2).

### Effect of CNP on cAMP levels in cumulus cells and oocytes of COCs

**Figure 3:**
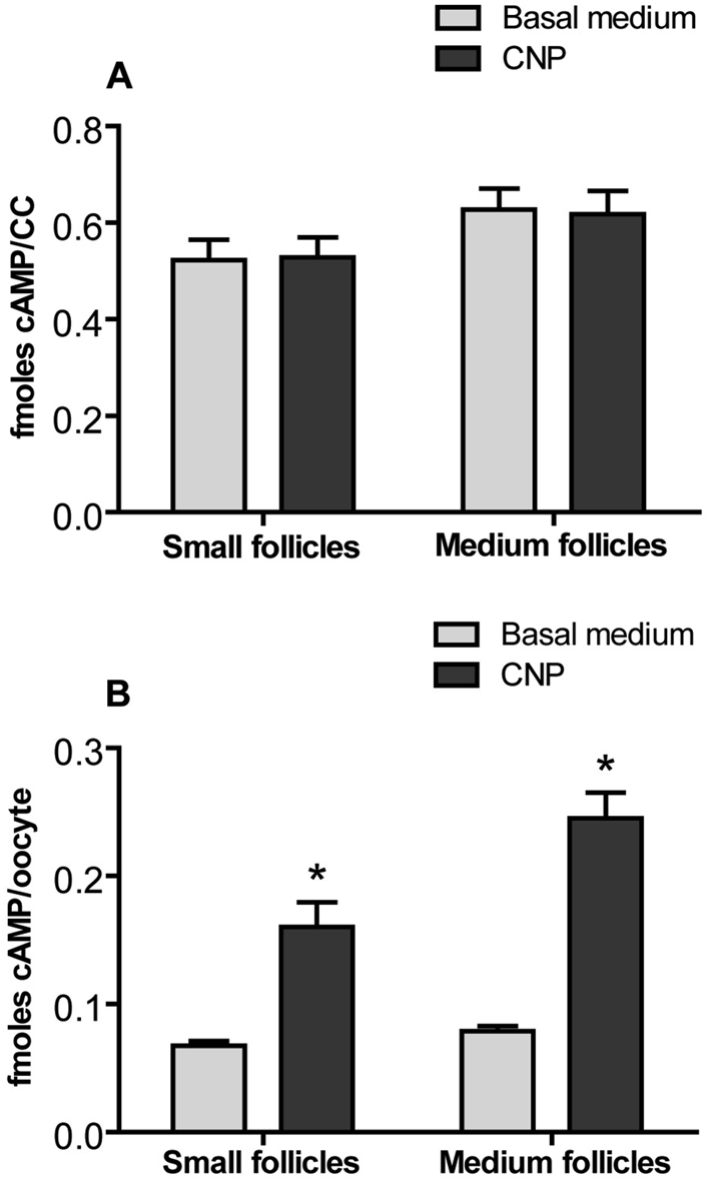
Effect of CNP on the levels of cAMP in bovine cumulus cells and oocytes. COCs derived from small follicles (SFs) and medium follicles (MFs) were cultured for 6 h in basal medium without or with 200 nM CNP. The levels of cAMP in bovine cumulus cells and oocytes were detected at the end of culture. (**A**) The levels of cAMP in bovine cumulus cells. (**B**) The levels of cAMP in bovine oocytes. Graphs show the mean ± SEM values of four independent experiments; The asterisks represent significant differences between the two groups with the same size follicle (*P* < *0.05*).

To determine whether CNP can sustain higher levels of cAMP in oocyte, COCs from SFs and MFs were cultured for 6 h in basal medium supplementated without or with 200 nM CNP. As shown in Fig. 3, CNP treatment significantly improved the cAMP levels in oocytes (*P* < *0.05*), but not in cumulus cells regardless of follicle size, compared to the control group (no CNP treatment) (*P* < *0.05*) (Fig. 3).

### Effect of CNP on cGMP /cAMP levels in cumulus cells and oocytes of COCs via activation of NPR2

**Figure 4:**
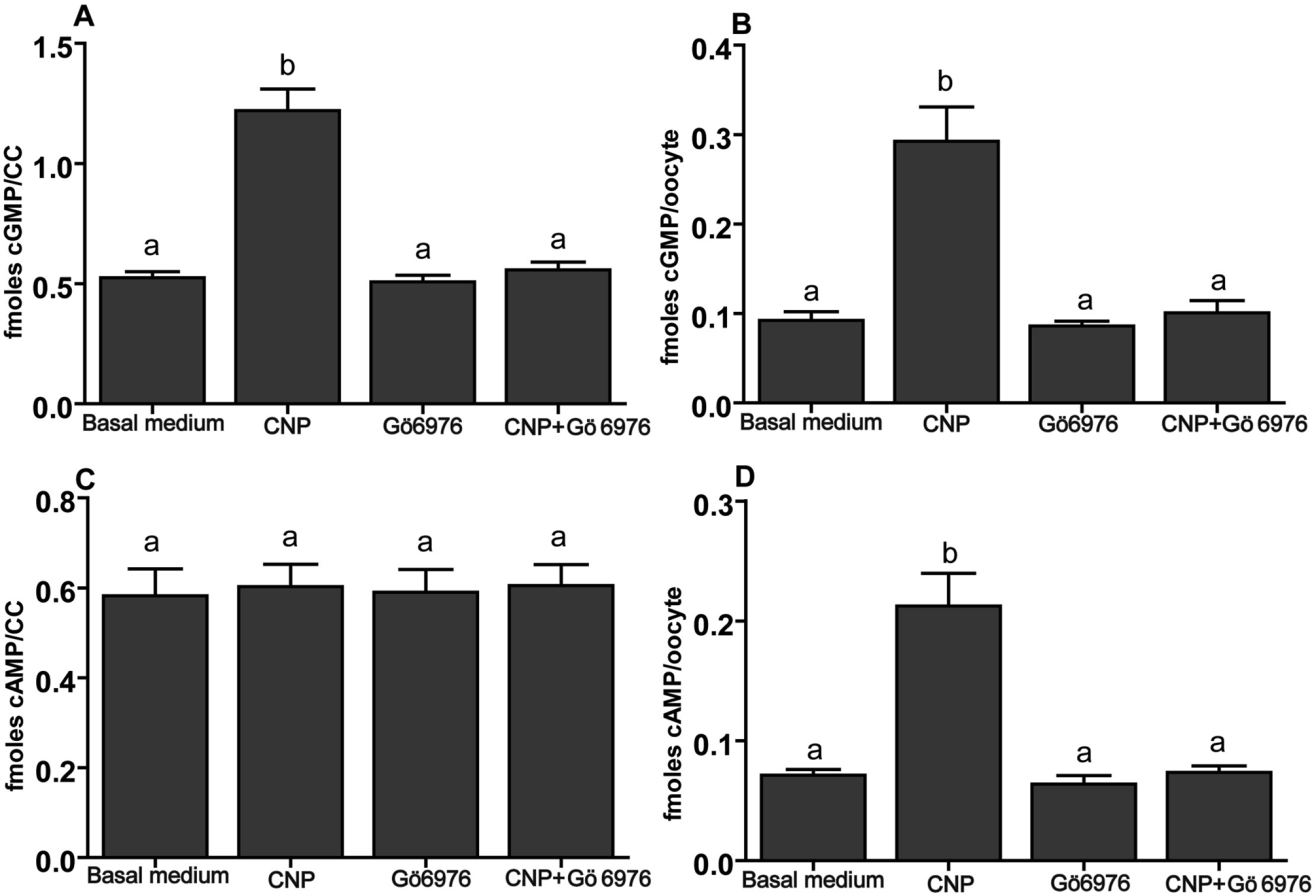
Inhibition of NPR2 abolishes the effect of CNP on the levels of cGMP/cAMP in bovine cumulus cells and oocytes. COCs derived from ovarian antral follicle were cultured in basal medium in the absence or presence of 200 nM CNP and/or 5 μM Gö6976, a competitive inhibitor of NPR2 (Basal medium, CNP, Gö6976, and CNP + Gö6976, respectively) for 6 h. The levels of cGMP/cAMP in bovine cumulus cells and oocytes were detected at the end of culture. (**A**) The levels of cGMP in bovine cumulus cells. (**B**) The levels of cGMP in bovine oocytes. (**C**) The levels of cAMP in bovine cumulus cells. (**D**) The levels of cAMP in bovine oocytes. Graphs show the mean ± SEM values of four independent experiments; Values indicated by different letters are significantly different (*P* < *0.05*).

As shown in Figs. 2 and 3, CNP elevated the levels of cGMP in cumulus cells and oocyte, as wellas the levels of cAMP in oocyte, suggesting that CNP may activate NPR2. To test this possibility, the NPR2-relative specific, but high-efficient competitive inhibitor Gö6976 (5 μM)^11^ together with CNP (200 nM) was added to the basal medium (CNP + Gö6976) during in vitro culture. As shown in Fig. 4, compared to basal medium group, the increased level of cGMP in cumulus cells and oocyte and cAMP in oocyte was no longer observed. Furthermore, compared to CNP-only treatment, the level of cGMP in cumulus cell and oocyte, and cAMP in oocyte was significantly decreased (*P* < 0.05) when COCs were treated by CNP and Gö6976 simultaneously (Fig. 4).

### Effect of CNP pre-treatment on bovine oocyte development

To investigate the effect of CNP pre-treatment on developmental competence of bovine oocyte derived from follicles of different sizes. A novel IVM system based CNP pretreatment was used, in which bovine oocytes were pre-treated with 200 nM CNP in TCM199 medium supplemented with 0.4% BSA for 6 h, then subjected to IVM and fertilized after 28 h IVM^11^ (Xi et al. 2018). Results showed that this IVM system based CNP pretreatment significantly improved the cleavage and blastocyst rates of oocytes collected from MFs *(P* < *0.05*), but not from SFs (*P* > *0.05*) following in vitro fertilization (IVF) (Table. 1). In addition, the cell numbers of per blastocyst, considered a marker of embryo quality, was significantly higher in CNP based MFs IVM group compared to other IVM groups (*P* < *0.05*) (Table. 1). These findings indicate that although CNP pre-IVM treatment enhanced the developmental potencial of oocytes obtained from MFs, but had no effect on the developmental comptence of oocytes obtained from SFs (*P* > *0.05*).

**Table 1:** Effects of the novel IVM system based on CNP-pretreatment on developmental competence of bovine oocyte derived from follicles of different sizes.

Group	Oocytes (N)	Cleavage rate (%)	Blastocyst rate (%)	Total cell numbers of blastocyst (N)
Conventional IVM (SFs)	187	52.4 ± 3.0^c^ (N = 98)	11.8 ± 1.1^c^ (N = 22)	104.3 ± 4.8^b^ (N = 20)
CNP-pretreatment based IVM (SFs)	178	56.7 ± 4.8^c^ (N = 101)	14.0 ± 1.4^c^ (N = 25)	106.3 ± 6.2^b^ (N = 22)
Conventional IVM (MFs)	188	73.0 ± 4.0^b^ (N = 138)	23.4 ± 2.7^b^ (N = 44)	108.2 ± 5.3^b^ (N = 23)
CNP-pretreatmentbased IVM (MFs)	176	86.9 ± 2.7^a^ (N = 153)	38.1 ± 3.0^a^ (N = 67)	127.1 ± 6.3^a^ (N = 25)

Data are expressed as mean ± SEM of four independent experiments. Blastocyst rate is calculated based on the number of the oocytes. Different superscripted letters in the same column indicate a statistically significant difference (*P* < *0.05*).

## Discussion

It is generally accepted that high levels of intra-oocyte cAMP are essential for maintaining the meiotic arrest of mammalian oocytes in vivo^17,18^. In addition to the generation of cAMP from mural granulosa and/or cumulus cells, the oocyte itself can also produce cAMP due to activation of the constitutively active heterotrimeric G protein (Gs)-linked receptors GPR3 or GPR12, which primarily induces meiotic arrest via protein kinase A (PKA)-mediated phosphorylation of the proteins that regulate cyclin dependent protein kinases^19^. An inability to suppress cAMP phosphodiesterase PDE3A activity in mouse oocytes would reduce the levels of cAMP, resulting in meiotic resumption in a gonadotropin-independent manner^20,21^. Moreover, a previous report has indicated that the transfer of cGMP from mouse cumulus cells to oocytes via gap junctions is a critical factor in the inhibition of PDE3A^22^. More importantly, it has already been demonstrated that CNP stimulates cGMP production in cumulus cells by activating NPR2, and cGMP diffuses into the oocyte from companion cumulus cells via gap junctions and inhibits oocyte PDE3A activity and cAMP hydrolysis, therebying maintaining meiotic arrest in mouse oocytes^10^. Hence, the CNP induced-cGMP in cumulus cells are crucial for maintaining sufficient intra-oocyte cAMP to inhibit oocyte meiotic resumption in the mice. It has been demonstrated that PDE3A is predominantly expressed in bovine oocytes^23^. Additionally, high levels of cAMP sustained by inhibiting PDE3A activity, can transiently prevent the meiotic resumption of denuded bovine oocytes in vitro^20,24^. In particular, previous reports demonstrated that a PKA stimulator blocked meiotic maturation of bovine oocytes, whereas a PKA inhibitor interfered with the meiotic arrest of bovine oocytes induced by forskolin or cilostamide^25,26^. These findings also support the view that high levels of cAMP sustained in oocytes also plays a key role in the maintenance of meiotic arrest in bovine oocytes^27^. Notably, a recent study demonstrated that in addition to inducing cGMP generation in CCs, CNP can also activate intra-oocyte cGMP production via NPR2 localized in oocyte membranes, sustaining sufficient levels of cAMP to maintain meiotic arrest of bovine oocytes, which is distinct from that known in mouse^11^. Up to date, the research papers published revealed the mechanism underlying the CNP-induced meiotic arrest, primarily using mixed population of oocytes isolated from follicles with a diameter 2–8 mm^11,12^. However, the effect of CNP on meitic arrest in bovine oocytes isolated from SF with a diameter < 3.0 mm remain still unclear. Our data confirmed that CNP not only sustained meiotic arrest in bovine oocytes derived from SF with a diameter < 3.0 mm, but also increased the levels of cGMP in both CCs and oocyte, and the levels of cAMP in oocytes, indicating that CNP can also induce cGMP generation in bovine COCs from SF via NPR2 activation and thus inhibit intra-oocyte cAMP hydrolysis and meiotic resumption. Overall, these findings suggest that the high levels of cAMP and cGMP sustained by CNP/NPR2 signaling pathway plays a key role in maintaining meiotic arrest in bovine oocytes.

Currently, bovine oocytes matured in vitro are developmentally compromised. The low efficiency of oocyte IVM technology limits the exploration and utilization of oocyte sources from cattle with desirable traits. Extensive studies using meiotic inhibitors have been conducted to optimize the bovine IVM system; nevertheless, the role of these meiotic inhibitors in enhancing the development of bovine oocytes remains controversial^28–31^. Notably, several studies have demonstrated that CNP maintains meiotic arrest of immature oocytes after removal from antral follicles in different animal species^11,32,33^. These finding suggest CNP could be used as an effective and safe factor to optimize in vitro maturation culture system, thereby facilitating the developmental competence of oocytes matured in vitro. Indeed, the potential of CNP to improve oocyte competence and embryo quality in mammals has alreadly been demonstrated in large animal and human models^11,33–35^. Among these models, a novel IVM system based on CNP-pretreatment, which includes a pre-IVM phase for 6 h using 200 nM CNP, followed by a extended IVM phase for 26–28 h, has been found to significantly promoted cytoplasmic maturation in bovine oocytes from follicles with a diameter 2–8 mm, as reflected by an improved developmental competence^11^. Similarly, our results showed that the novel IVM system based on CNP-pretreatment significantly improved the developmental comptence of bovine oocytes from MFs with a diameter 3–8 mm. The mechanisms by which CNP improved bovine oocyte cytoplasmic maturation might be due to elevated levels of maternal mRNA and proteins during meiotic arrest in vitro, resulting in an increased rate of embryo development beyond the “maternal-to-embryonic transition” stage^36^.

Oocyte quality is associated with follicle size^15^. The present study reported a significantly higher cleavage and blastocyst rates in MFs oocytes than those of SF-derived oocytes, which was similar with previous findings^16,37^. At present, the mixed population of bovine oocytes isolated from MFs with a diameter 3–8 mm were commonly used for in vitro embryo production^38–40^. However, whether the novel IVM system based on CNP-pretreatment can improve the developmental comptence of bovine oocytes from SF with a diameter < 3 mm is still unkown. The present study confirmed that CNP pre-IVM treatment had no effect on the developmental comptence of bovine oocytes obtained from SFs, suggesting a short period of meiotic arrest prior to IVM using CNP fails to adequately support cytoplasmic maturation of bovine oocyte from SFs with a diameter < 3 mm. Oocyte developmental competence, which is acquired gradually during oocyte growth and maturation in the ovarian follicle, is regulated by a number of signals molecules as well as cumulus cells^41–43^. Furthermore, previous studies have shown that COCs derived from SFs lack the ability to respond to regulatory and specific ligands because of an immature signaling capacity^44–46^. Thus, we hypothesized that during CNP pre-IVM period, the signaling networks in oocytes and surrounding cumulus cells from SFs, which participate in the development of oocyte competence, might be insufficient for promoting oocyte cytoplasmic maturation compared to those in oocytes and surrounding cumulus cells from MFs. Therefore, to develop more effective culture systems, further studies should focused on how to enhance the signaling capacity of COCs from SFs by supplementing with some regulatory factor during CNP pre-IVM period.

In summary, CNP increased the levels of cGMP in both cumulus cells and the oocyte from different follicle size through activation of NPR2, thereby sustaining meiotic arrest in bovine oocyte. In addition, CNP pre-IVM treatment enhanced the developmental potencial of bovine oocytes obtained from MFs, but had no effect on the developmental competence of bovine oocytes obtained from SFs.

## Materials and methods

Our research was approved by the Laboratory Animal Resource Center of Inner Mongolia University for the Nationalities and was performed in accordance with the Animal Care and Use Statute of China.

### Chemicals

Unless otherwise stated, all chemicals and reagents used were purchased from Sigma-Aldrich (St. Louis, MO, USA). HEPES-buffered tissue culture medium 199 (HTCM199, 12340-030) and TCM199 (11150-059) were purchased from Life Technologies. Follicle stimulating hormone (FSH) and luteinizing hormone (LH) were purchased from Bioniche (Belleville, Canada).

### Collection of cumulus cell-oocyte complexes (COCs) and culture

COCs were collected from the antral follicles of bovine ovaries obtained from an local abattoir. Follicles were dissected with scissors at room temperature, measured under a stereomicroscope and separated into two groups according to diameter: small follicles, < 3 mm; medium, 3–8 mm. COCs were liberated by carefully rupturing the follicles^16^. The COCs with more than three cumulus cells layers and a uniform ooplasm were selected and washed in the basal culture medium (TCM199 with Earle’s salts supplemented with 0.4% fatty acid-free BSA) for subsequent study^12^. To examine the effects of CNP on oocyte meiotic arrest and the levels of cAMP and cGMP in bovine COCs, the COCs were cultured in basal medium with or without 200 nM CNP for 6 h^11^. All COCs were cultured at 38.5 °C under 5% CO2 in humidified atmosphere.

### The assessment of oocyte meiosis arrest

To evaluate meiosis arrest in bovine oocytes, after 6 h of culture, cumulus cells were removed from COCs by using 5 mg/ml hyaluronidase in TCM199 medium and repeated pipetting, Denuded oocytes were fixed in 4% paraformaldehyde for 30 min at room temperature, permeabilized using 0.1% Triton-100 in PBS for 20 min, and stained using 4′,6-diamidino-2-phenylindole (DAPI). Oocytes with germinal vesicles (GV; meiotic arrest) were detected using a fluorescence microscope (Eclipse Ci-s; Nikon).

### Measurement of cGMP and cAMP levels in cumulus cells and oocytes

The cGMP and cAMP levels in bovine CCs and oocyte were detect as described previously^11^ (Xi et al. 2018). Briefly, after 6 h of culture, groups of ~ 50 COCs were collected. To obtain both the cumulus cells and the associated oocytes, the COCs were mechanically dissociated by manual pipetting with a small bore glass pipette in TCM199 containing 2 mM IBMX. After a rapid wash with PBS containing 2 mM IBMX, the CCs and DOs were transferred to 100 ml of 0.1 M HCl for 25 min to lyse the cells and the tubes were then centrifuged at 13,000 g for 5 min, the supernatant was transferred to new tubes and stored at − 80 °C until assayed using the cGMP and cAMP enzyme-linked immunosorbent assay kit, according to the manufacturer's instructions. Eeach experiment was repeated independently at least four times.

### In vitro maturation, fertilization and culture

Conventional in vitro maturation (IVM) was performed as previously described^47^. Briefly, COCs were matured in 500 µl TCM199 supplemented with 10 µg/ml FSH, 1 µg/ml LH and 1 µg/ml estradiol for 24 h in 4-well culture plates (Nunclon; VWR, Bridgeport, NJ, USA) at 38.5 °C under 5% CO_2_ in humidified atmosphere. To evaluate the effect of CNP pretreatment on the developmental potential of oocyte derived from follicles of different sizes, oocytes were matured in basal culture medium with 200 nM CNP for 6 h, then subjected to an extended IVM culture for 28 h^11^ (Xi et al. 2018). After maturation, the COCs were fertilized in BO medium for 6 h at 38.5 °C under 5% CO2 in humidified atmosphere. After in vitro fertilization (IVF), the presumptive zygotes were transferred to 100 μl CR1 culture medium droplets at 38.5 °C under 5% CO2 in humidified atmosphere. The culture medium was changed every 2 days during the culture period. The cleavage and blastocyst rates were recorded on day 2 and 7 after fertilization, respectively.

### Blastocyst cell number assessment

On day 7 of culture, blastocysts were fixed for 30 min with 4% paraformaldehyde in PBS, then stained for 10 min with Hoechst 33242 (10 µg/ml) in PBS containg 0.5% (v/v) Triton X-100. Embryos were mounted on slides and examined using a fluorescence microscope (Eclipse Ci-s; Nikon).

### Statistical analysis

All data are presented as the mean ± standard error of the mean. All proportional data were subjected to arcsine transformation before statistical analysis. All data obtained in the present study were assessed using one-way analysis of variance (ANOVA) followed by Duncan's test using SPSS version 12.0, (SPSS, Chicago, IL, USA). Statistical significance was defined as *P* < *0.05*.
